# A preoperative nomogram predicts prognosis of patients with hepatocellular carcinoma after liver transplantation: a multicenter retrospective study

**DOI:** 10.1186/s12885-021-07938-x

**Published:** 2021-03-16

**Authors:** Dabing Huang, Yinan Shen, Wei Zhang, Chengxiang Guo, Tingbo Liang, Xueli Bai

**Affiliations:** 1 Department of Hepatobiliary and Pancreatic Surgery, the First Affiliated Hospital, School of Medicine, Zhejiang University, 79 Qingchun Road, Hangzhou, 310003 Zhejiang China; 2 Zhejiang Provincial Key Laboratory of Pancreatic Disease, the First Affiliated Hospital, School of Medicine, Zhejiang University, Hangzhou, 310003 Zhejiang China; 3 Zhejiang Provincial Innovation Center for the Study of Pancreatic Diseases, Hangzhou, 310003 Zhejiang China

**Keywords:** Hepatocellular carcinoma, Liver transplantation, Risk factors, Nomogram

## Abstract

**Background:**

Although criteria for liver transplantation, such as the Milan criteria and Hangzhou experiences, have become popular, criteria to guide adjuvant therapy for patients with hepatocellular carcinoma after liver transplantation are lacking.

**Methods:**

We collected data from all consecutive patients from 2012 to 2019 at three liver transplantation centers in China retrospectively. Univariate and multivariate analyses were used to analyze preoperative parameters, such as demographic and clinical data. Using data obtained in our center, calibration curves and the concordance Harrell’s C-indices were used to establish the final model. The validation cohort comprised the patients from the other centers.

**Results:**

Data from 233 patients were used to construct the nomogram. The validation cohort comprised 36 patients. Independent predictors of overall survival (OS) were identified as HbeAg positive (*P* = 0.044), blood-type compatibility unmatched (*P* = 0.034), liver transplantation criteria (*P* = 0.003), and high MELD score (*P* = 0.037). For the validation cohort, to predict OS, the C-index of the nomogram was 0.874. Based on the model, patients could be assigned into low-risk (≥ 50%), intermediate-risk (30–50%), and high-risk (≤ 30%) groups to guide adjuvant therapy after surgery and to facilitate personalized management.

**Conclusions:**

The OS in patients with hepatocellular carcinoma after liver transplantation could be accurately predicted using the developed nomogram.

## Background

The incidence of liver cancer, despite showing some slow down, increased by 2–3% annually from 2007 to 2016 [1]. R0 resection and liver transplantation (LT) are regarded as the key methods for the complete remission of liver tumors [2, 3]. LT replaces the diseased liver while simultaneously providing complete oncological resection, which represents a predisposing factor in greater than 90% of patients with hepatocellular carcinoma (HCC) [4].

Over the last 20 years, various criteria incorporating preoperative variables have been formulated to limit the selection of patients with HCC for LT [5–8]. In 1996, the Milan criteria (MC) were introduced and have been used widely ever since. The MC limited LT to patients with a single tumor with a diameter up to 5 cm diameter or up to three tumors, none of which is larger than 3 cm [5]. Twelve years later, the Hangzhou Experience (HZ) was established by our group, which had wider surgical indications than the MC and was more suitable for Asian populations [6]. Thereafter, criteria for postoperative guidance were lacking until Agopian et al. reported a prognostic system that could accurately predict HCC recurrence after LT [4]. Unfortunately, this model focused on HCC recurrence and was constructed on Caucasian population data. Therefore, we established this large, multiple-center retrospective study comprising Chinese patients with HCC who underwent LT at three liver transplantation centers in China. The study aimed to formulate a nomogram to predict the long-term survival of these patients, to guide adjuvant therapy, and facilitate personalized management.

## Methods

### Patients participating in the study

The present study included patients who were diagnosed with HCC and underwent LT from 2012 to 2019 at three liver transplantation centers in China. The three centers comprised: the First Affiliated Hospital of Zhejiang University School of Medicine in Hangzhou, Tianjin First Center Hospital in Tianjin, and the Third Affiliated Hospital of Sun Yat-sen University in Guangzhou. These institutions perform high volumes of liver transplantation. We only included patients who underwent LT and who had histopathologically confirmed HCC. The inclusion criteria comprised: no history of other malignant tumors; and no surgical procedures other than LT. If a patient died within 30 days of LT, they were excluded from this study.

### Imaging and pathological analysis

Magnetic resonance imaging (MRI) or computed tomography (CT) were used to determine the extent of pre-transplantation disease. A radiologist who was blinded to the patients’ clinical records analyzed all the images retrospectively. The tumors of patients with HCC were classified as within the Milan criteria, beyond the Milan criteria but the within Hangzhou criteria, or exceeding the Hangzhou criteria. All liver explants were reviewed by an experienced pathologist and categorized based on lymph node involvement, presence of macro- and microvascular invasion, histological grade and differentiation, distribution, size, and tumor number. The tumor-node-metastasis (TNM) stage of the patients with HCC in our cohort was based on the America Joint Committee on Cancer TNM staging system (8th edition) [9].

### Patient follow-up

The patients were subjected to follow-up once every 2 weeks in the first 3 months, every 4 weeks in the first 1 year, and then once every 3 months. A detailed clinical history was recorded and a complete physical examination was performed at each follow-up visit. This study was censored on Dec. 31st, 2019.

### Statistical analysis

SPSS 19.0 for Windows (IBM Corp., Armonk, NY, USA) and Prism 6 for Windows version 6.01 (GraphPad Software, San Diego, CA, USA) were used to identify risk factors. Clinical findings were used to group the categorical variables, and the groups were decided upon before modeling. The chi-squared test or Fisher’s exact test were used to compare all the results. To compare continuous variables, we used Student’s t-test or the Mann-Whitney U test for variables with abnormal distributions. The log-rank test was used to compare survival curves that had been constructed using the Kaplan-Meier method. Multivariate analysis was executed using Cox regression analysis.

The results of multivariate analysis formed the basis to construct the nomogram, using the rms26 package in R version 3.3.2 (http://www.r-project.org/). A backward step-down selection process with the Akaike information criterion was used to perform the final model selection [10]. The concordance index (C-index) was used to measure the performance of the nomogram. A comparison of the nomogram-predicted versus the observed Kaplan-Meier estimates of survival probability formed the basis of the assessment of nomogram performance. During nomogram validation, the established nomogram was used to calculate the total points for each patient from the validation cohort. Subsequently, the total points were used as a factor in the Cox regression analysis in this cohort. Finally, the regression analysis was used to derive the C-indices and the calibration curves. Statistical significance was accepted at a *P* value less than 0.05.

## Results

### Characteristics of the patients

Three liver transplantation centers provided retrospective data for 281 patients. Twelve patients in our center died within 30 days after surgery, including 7 cases of postoperative acute immune rejection, 3 cases of massive postoperative bleeding, and 2 cases of severe lung infection. Other patients in our center were used as the training cohort (*n* = 233) and the patients in the remaining centers comprised the validation cohort (*n* = 36). There was a near 6:1 proportion of patients between two groups. The patients’ baseline characteristics are shown in Table 1.

**Table 1:** Patient Baseline Characteristics by Cohort

	Training cohort (***n*** = 233)		Validation cohort 1 (***n*** = 16)		Validation cohort 2 (***n*** = 20)	
	No. of patients	%	No. of patients	%	No. of patients	%
**Sex**						
Male	208	89.3	15	93.75	17	85.0
Female	25	10.7	1	6.25	3	15.0
**Age, years**						
Median	53.0		53.0		53.5	
IQR	48.0–60.0		47.3–61.8		47.8–62.0	
**LT criteria**						
Milan	95	40.8	14	87.5	14	70.0
Hangzhou	42	18.0	2	12.5	4	20.0
Over Hangzhou	96	41.2	0	0	2	10.0
**Smoke**						
Yes	128	54.9	13	81.2	12	60.0
No	105	45.1	3	18.8	8	40.0
**Alcohol**						
Yes	77	33.0	10	62.5	8	40.0
No	156	67.0	6	37.5	12	60.0
**BMI**						
Median	22.8		23.9		21.5	
IQR	21.1–25.1		21.2–25.6		19–22.9	
**AFP, ng/mL**						
Median	13.9		31.1		174.6	
IQR	3.4–256.8		6.2–426.1		6.9–2915.2	
**CEA, ng/ml**						
Median	2.6		1.3		2.8	
IQR	1.8–4.1		1.0–1.9		1.8–3.8	
**TB,** μmol**/mL**						
Median	26.0		25.1		26.1	
IQR	15–55.5		10.4–120.8		15.7–76.6	
**Albumin, g/L**						
Median	34.0		37.9		35.8	
IQR	30.4–38.3		34.5–40.7		18.3–44.1	
**ALT, U/L**						
Median	34.0		33.5		35.8	
IQR	20.5–60.5		21.5–62.8		18.3–44.1	
**AST, U/L**						
Median	45.0		32.0		47.6	
IQR	31–80		24.3–110.3		26.2–74.1	
**Hypertension**						
Yes	40	17.2	2	12.5	5	25.0
No	193	82.8	14	87.5	15	75.0
**Diabetes**						
Yes	26	11.2	2	12.5	4	20.0
NO	207	88.8	14	87.5	16	80.0
**Ascites**						
No	142	60.9	9	56.3	13	65.0
Mild	70	30.0	6	37.5	1	5.0
Severe	21	9.1	1	6.3	6	30.0
**HBeAg**						
Positive	56	24.0	15	93.8	17	85.0
Negative	177	76.0	1	6.2	3	15.0
**Blood type**						
Matched	189	81.1	15	93.8	19	95.0
Unmatched	44	18.9	1	6.2	1	5.0

### Overall survival (OS)-related prognostic factors

Univariate and multivariate Cox regression analyses were used to assess the demographic, clinical, and biochemical parameters of the training cohort to identify independent risk factors associated with OS. The results revealed that OS was significantly associated with the presence of ascites, HbeAg positive, blood-type compatibility unmatched, liver transplantation criteria, and high model for end-stage liver disease (MELD) (Table 2). Upon multivariate analysis, HbeAg positive, blood-type compatibility unmatched, liver transplantation criteria, and a high MELD score were suggested as independent OS-related risk factors (Table 2).

**Table 2:** Univariate and multivariate analyses of factors associated with overall survival

	Overall survival					
	Univariate analysis			Multivariate analysis		
	HR	95% CI	*P* value	HR	95% CI	*P* value
Age, years	1.009	0.984–1.036	0.475			
Sex (male/female)	1.120	0.515–2.439	0.775			
Smoking (yes/no)	1.266	0.801–2.000	0.313			
Drinking (yes/no)	1.472	0.926–2.342	0.102			
BMI, kg/m^2^	0.999	0.995–1.003	0.613			
Hypertension (absence/ presence)	1.211	0.665–2.204	0.531			
Diabetes (absence/ presence)	1.339	0.687–2.610	0.391			
Ascites (absence/ presence)	1.473	1.079–2.011	**0.015**	1.132	0.802–1.598	0.480
HbsAg (negative/positive)	1.535	0.667–3.534	0.314			
HbeAg (negative/positive)	1.696	1.056–2.725	**0.029**	1.640	1.014–2.651	**0.044**
Blood-type compatibility (matched/unmatched)	2.252	1.378–3681	**0.001**	1.733	1.034–2.880	**0.034**
Liver transplantation criteria	1.678	1.279–2.203	**0.000**	1.536	1.157–2.037	**0.003**
MELD score	1.058	1.019–1.100	**0.004**	1.044	1.003–1.088	**0.037**
Child-Pugh classification	1.001	0.895–1.121	0.980			
WBC, 10*9/L	1.036	0.996–1.077	0.078			
HGB, g/L	1.004	0.996–1.013	0.313			
PLT, 10*9/L	1.002	0.999–1.005	0.112			
INR	0.574	0.295–1.119	0.103			
Serum CRE, μmol/L	0.998	0.994–1.002	0.343			
Serum AFP, ng/mL	1.000	1.000–1.000	0.082			
Serum CEA, ng/mL	0.944	0.850–1.049	0.283			
Serum CA125, U/L	1.000	0.999–1.001	0.613			
Serum ALT, U/L	1.001	0.999–1.003	0.279			
Serum AST, U/L	1.000	1.000–1.000	0.416			
Serum ALP, U/L	0.999	0.997–1.001	0.190			
Serum GGT, U/L	0.999	0.997–1.001	0.114			
Serum TB, μmol/L	0.999	0.997–1.002	0.654			
Serum ALB, g/L	1.005	0.966–1.046	0.795			

*CI* confidence interval, *HR* hazard ratio, *BMI* body mass index, *MELD* model for end-stage liver disease, *WBC* white blood cell, *HGB* hemoglobin, *PLT* platelets, *INR* international normalized ratio, *CRE* creatinine, *AFP* alpha fetoprotein, *CEA* carcino-embryonic antigen, *CA125* carbohydrate antigen 125, *ALT* alanine aminotransferase, *AST* aspartate aminotransferase, *ALP* alkaline phosphatase, *GGT* gamma-glutamyl transpeptidase, *TB* total bilirubin, *ALB* albumin

### OS-associated risk factors-based prognostic nomogram

**Fig. 1:**
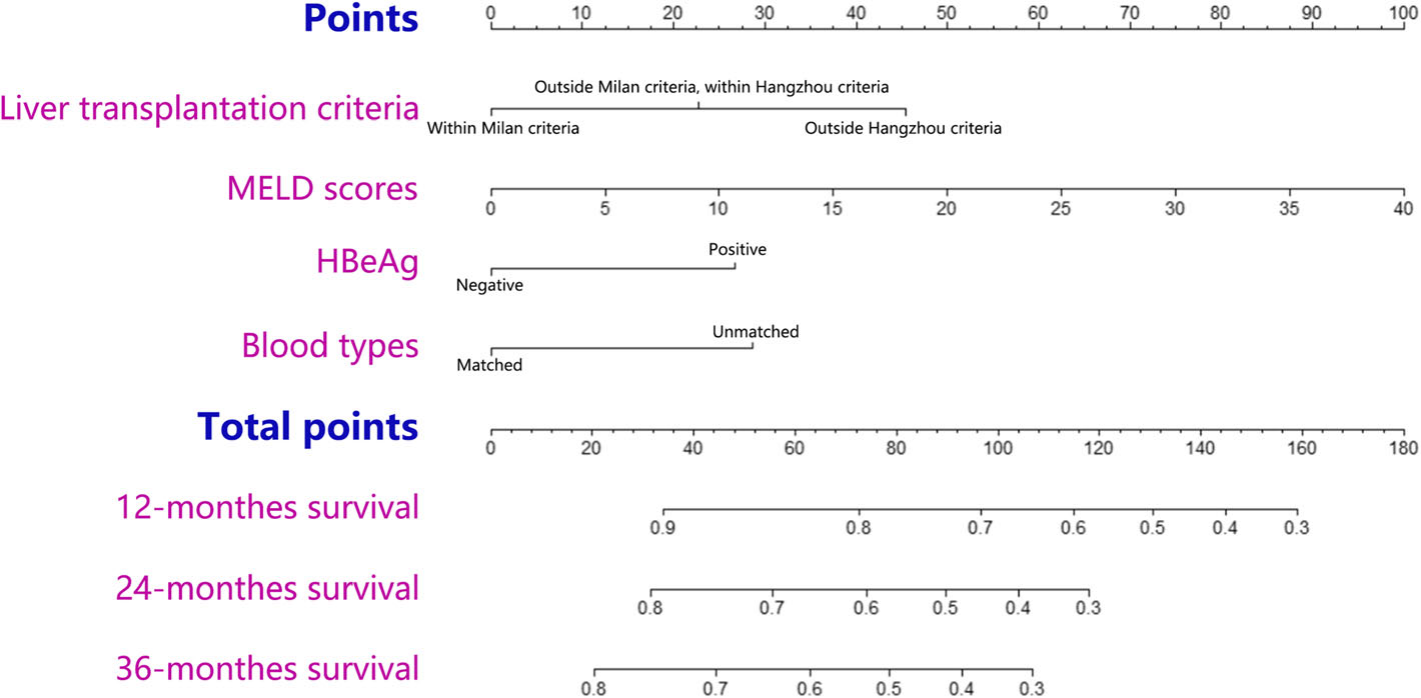
Predictive nomogram for OS in patients with HCC after LT

The four identified independent risk factors for OS were used to construct a nomogram to predict the survival of patients with HCC after LT (Fig. 1). The odds ratio (OR) was used to weight the assignment of points to each factor. The total score was then used to calculate the survival probability at specific times (1, 2 and 3 years post-LT). For example, a patient with HCC after LT within the MC criteria (0 point), with a MELD score of 20 (50 points), HbeAg positive (26 points), and blood-type compatibility matched (0 points) would have a total score of 76 points, which corresponded to survival probabilities of 79, 58, and 51% at 1, 2, and 3 years, respectively.

**Fig. 2:**
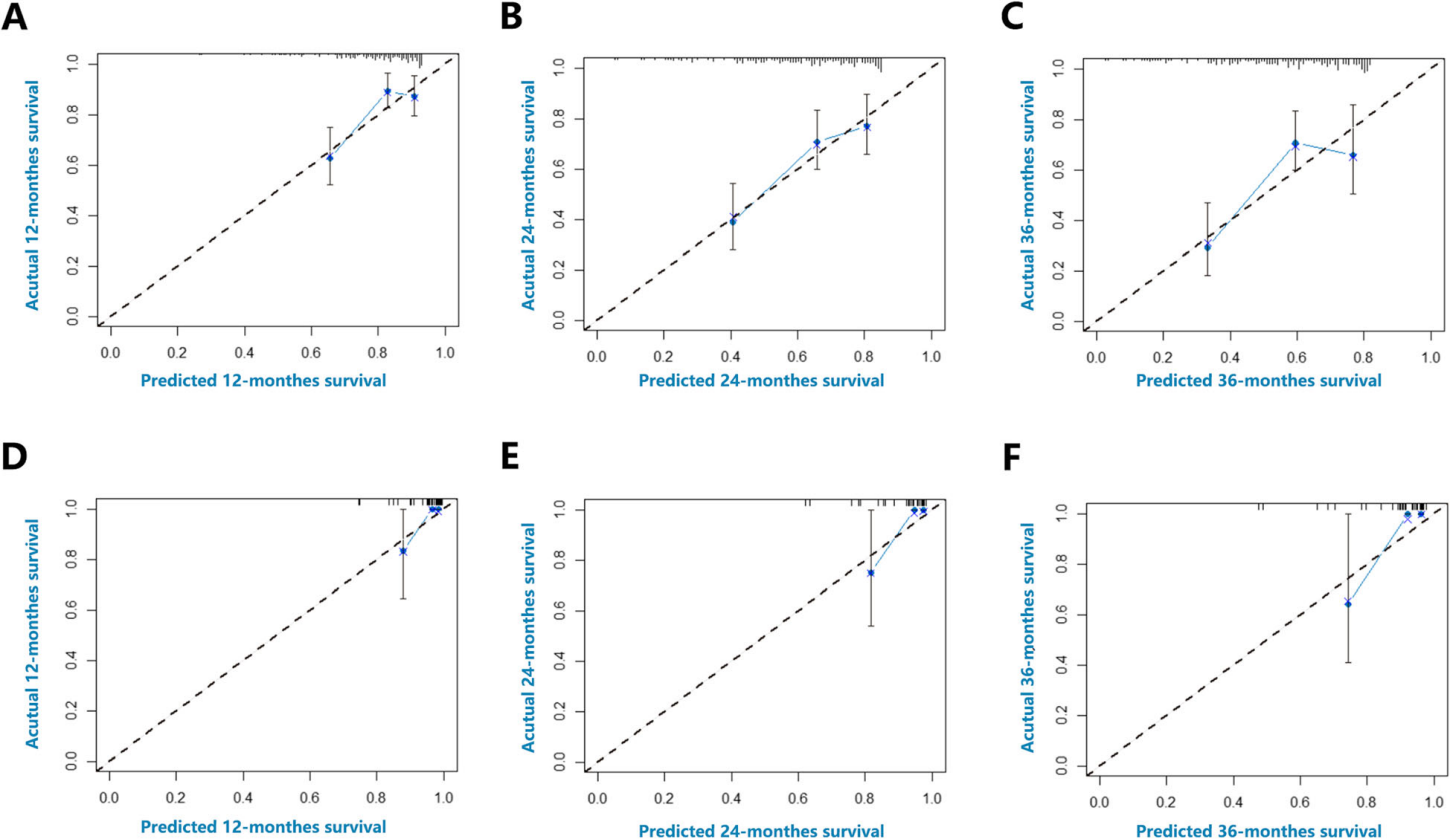
Calibration curves for predicting patients’ survival at (**a**) 12 months, (**b**) 24 months, and (**c**) 36 months in the training cohort and at (**d**) 12 months, (**e**) 24 months, (**f**) 36 months in the validation cohort

Using the nomogram, the training cohort received a C-index for the prediction of OS of 0.677 (95% CI, 0.606–0.748; Fig. 2, upper panel), and the C-index was 0.874 for the validation cohort (95% CI, 0.753–0.995; Fig. 2, lower panel).

### Risk groups

Three risk groups were identified using the risk scores calculated using the nomogram (Table 3): 1) The low-risk group (total points ≤ 79 and a predicted survival rate ≥ 50%), with a mean predicted mean survival rate = 68.2%; 2) The intermediate-risk group (total points 79–107 and a predicted survival rate of 30–50%), with a mean predicted survival rate = 39.6%; and 3) the high-risk group (total points ≥ 107 and a predicted survival rate ≤ 30%), with a mean predicted survival rate = 20.1%.

**Table 3:** Risk groups based on the predicted nomogram

Group	Total Points	Predicted Survival Rate	Predicted Mean Survival Rate	Observed Survival Rate
High-risk group	> = 107	<=30%	20.1%	28.0% (7/25)
Intermediate-risk group	79–107	30–50%	39.6%	48.1% (25/52)
Low-risk group	< = 79	> = 50%	68.2%	78.2% (122/156)

**Fig. 3:**
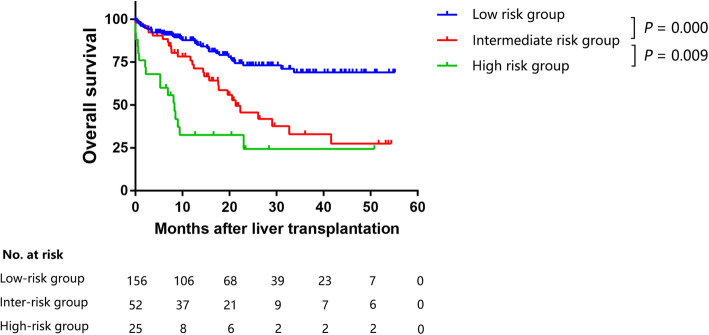
Cumulative survival rates in the different risk groups (the training cohort)

The observed survival rate conformed completely with the mean predicted survival rate. Among the risk groups there were significant differences in survival (*P*<0.0001). Figure 3 shows the cumulative patient survival rates for the three groups. The low risk group had significantly better survival than the intermediate-risk group (*P* = 0.000). There was also a significant difference between the high-risk and intermediate-risk groups (*P* = 0.009).

## Discussion

Whether LT is the best treatment option for HCC is a matter of debate [6]. However, currently, LT is widely accepted as the best treatment modality. The introduction of the Milan criteria (MC) represented a breakthrough in the standardization of LT and the Hangzhou experiences (HZ) further broadened the indications for surgery. However, increasing evidence showed that these criteria are too conservative and only focus on the tumor status. In fact, whole body condition of patients and other parameters, such as liver function, radiological appearance, and epidemiological factors, can also affect the outcome of LT. The present study aimed to identify independent risk factors for prognosis in patients with HCC who received LT. These risk factors were then used to establish a nomogram for the presurgical prediction of OS.

During the construction of the nomogram, we did not try to replace the original standards, such as the MC or HZ criteria. We included these criteria and other parameters, including laboratory results, epidemiological factors, and the previous history of the patients, but not tumor status. As expected, multivariate analysis identified the LT criteria as one of the risk factors of prognosis. Patients with tumors within the MC criteria, beyond the MC but within the HZ criteria, or exceeding the HZ criteria were given different statistical weights. Patients with tumors exceeding the HZ criteria received 45 points and had the worst prognosis because of advanced tumor and poor patient status. Initially, the MELD score was designed to evaluate renal and hepatic dysfunction-induced organ failure. However, it has been well-validated to guide organ allocation policy and to identify patients who are at risk of death because of end-stage liver disease [11]. In general, organs with a high donor risk index (DRI) should be transplanted into patients with a low MELD score of 10–14 [12, 13]. However, subsequent data showed that patients with high MELD scores could also benefit from sub-optimal organ transplants [12–14]; therefore, we included the entire range of MELD scores into our model.

In this study, HbeAg positive was found a risk factor of OS. It was reported that the risk of HCC increased significantly when associated with a single positive HbeAg [15]. Other studies reported that persistent viremia might result in active liver disease after HbeAg seroconversion or fluctuating HBeAg status, which would increase the risk of HCC and liver cirrhosis in these patients [16, 17]. However, Chan et al. reported that compared with HBeAg positivity, HBV-DNA is a more important risk factor for HCC [18]. Unfortunately, the data on HBV-DNA was lacking in our study, which might have affected the accuracy of our nomogram. In addition, HbeAg positivity always occurs in the active phase of hepatitis. Most HCC are due to cirrhosis caused by HBeAg positive, and cirrhosis is the most important risk factor for HCC. Studies on the correlation between cirrhosis and HCC have shown that the forming procession of HCC is largely mediated by inflammation, which causes the cycle facts of cell death and regeneration and induces the repeated proliferation of liver cells [19]. The release of inflammatory factors and poor patient status will also affect the result of LT. Hepatitis B X protein (HBx) is encoded by HBV, and it has been demonstrated that HBx is closely linked to HBV-related HCC [20]. HBx plays a multifunctional role in common biochemical pathways by modulating the expression and activities of numerous genes, contributing to many pathological processes, including viral replication, gene transcription, signal transduction and protein degradation, which are ultimately linked to the occurrence and development of HCC [21, 22].

Studies have shown increased prevalence of antibody-mediated rejection and cholangitis, lower graft survival and hepatic artery thrombosis in ABO- unmatched LT compared with those in ABO-matched LT. These observations could explain the results of the present study. In addition, a meta- analysis also showed worse graft survival in ABO- unmatched LT compared with that in ABO- matched LT [23]. When the blood type of A, B and O are incompatible, the antibody of anti-A or B in recipient’s blood will combine with the antigen directly, which is on the vascular endothelial cells of graft, and produce an antigen-antibody complex, activate the complement system quickly. As a result, it will destroy the vascular plexus and small bile ducts in the graft, form the extensive thrombotic microangiopathy, cause a series of rejection reactions and complications. The above factors will reduce the survival rate of the graft [24].

This study has some limitations. First, our study included four clinical indicators and no biological ones. Second, despite having 269 patients in this study, the validation cohort had a relatively small sample size, which might have resulted in its high C-index (0.874). Similarly, due to the limited samples size in the high-risk group and there are only two long-term survivors after 20 months, it may cause our nomogram to have a certain deviation in the assessment of 24 months and 36 months. Larger-sized cohorts are required to further determine the clinical value of the nomogram. In addition, the C-index will become more accurate with the expansion of the sample size. Second, as stated above, the lack of HBV-DNA data might have influenced the application value of our model, and we hope to fix this problem in future.

## Conclusion

A novel nomogram for OS prediction of patients with HCC after LT was constructed based on four essential independent risk factors determined at diagnosis. This nomogram showed good performance, with a C-index = 0.874 for the external validation cohort. The model allowed us to classify patients with HCC after LT into three risk groups. The identified risks could be used to provide specific management strategies in the future.

## Data Availability

The datasets used and analysed during the current study available from the corresponding author on reasonable request.

## Abbreviations

LT: Liver transplantation
HCC: Hepatocellular carcinoma
MC: Milan criteria (MC)
HZ: Hangzhou Experience
MRI: Magnetic resonance imaging
CT: Computed tomography
TNM: The tumor-node-metastasis
OS: Overall survival
MELD: Model for end-stage liver disease
OR: Odds ratio
